# Effectiveness and Sustainability of Water Chlorination in Public Healthcare Services in Guatemala

**DOI:** 10.3390/tropicalmed11050111

**Published:** 2026-04-23

**Authors:** Paulina Garzaro, Carmen Castillo, Natalie Fahsen, Lucas Santos, Joyce Lu, Christiana Hug, Matthew Lozier, Douglas R. Call, Celia Cordón-Rosales, Brooke M. Ramay

**Affiliations:** 1 Center for Health Studies, Universidad del Valle de Guatemala, Guatemala City 01015, Guatemala; apgarzaro@uvg.edu.gt (P.G.); cecastillo@uvg.edu.gt (C.C.); nfahsen@uvg.edu.gt (N.F.); lfsantosep@gmail.com (L.S.); ccordon@uvg.edu.gt (C.C.-R.); 2 Department of Anthropology, Rutgers University, New Brunswick, NJ 08901, USA; jl2295@rwjms.rutgers.edu; 3 Division of Foodborne, Waterborne, and Environmental Diseases, National Center for Emerging and Zoonotic Infectious Diseases, Centers for Disease Control and Prevention (CDC), Atlanta, GA 30333, USA; tjv6@cdc.gov (C.H.); wfu2@cdc.gov (M.L.); 4 Oak Ridge Institute for Science and Education (ORISE), U.S. Department of Energy, Oak Ridge, TN 37830, USA; 5 United States Public Health Service, Silver Springs, MD 21203, USA; 6 Paul G. Allen School of Global Health, Washington State University (WSU), Pullman, WA 99164, USA; drcall@wsu.edu

**Keywords:** effectiveness, hand hygiene, healthcare facilities, participatory research, sustainability, water chlorination

## Abstract

Introduction: Healthcare-associated infections are a significant public health challenge, particularly in resource-limited settings. While hand hygiene is critical for infection prevention, contaminated water from hand hygiene stations (HHSs) in healthcare facilities (HCFs) may undermine infection control efforts. Chlorination can reduce microbial contamination in HHSs, ensuring that water intended for hygiene does not become an infection source. Methods: Water quality was monitored before and after the installation of on-site chlorine dispensers (CDs) in water tanks and HHSs of HCFs in Quetzaltenango, Guatemala, to evaluate their effectiveness in improving water quality. Focus groups were conducted to develop action plan proposals to ensure the intervention’s sustainability. Results: Before the intervention, 75% of HHS water samples tested positive for total coliforms, with 50% testing positive for presumptive extended-spectrum beta-lactamase (ESBL)-producing total coliforms, while 20% were *E. coli*-positive, with 50% presumptive ESBL-producing *E. coli*. After installing CD, 1% of samples were coliform-positive over a six-month period. Focus groups identified resource limitations and political barriers and proposed solutions such as developing operational manuals, strengthening inter-institutional relationships, and forming alliances with external organizations. Conclusion: Localized chlorination was successfully implemented using a community participatory approach to improve water quality in resource-limited HCFs. These findings have important implications for infection prevention and control.

## 1. Introduction

Healthcare-associated infections (HAIs) remain a major global public health concern, contributing significantly to patient morbidity, mortality, and increased healthcare costs [1]. The World Health Organization (WHO) underscores the critical importance of integrating infection prevention and control (IPC) measures involving water, sanitation, and hand hygiene (WASH) initiatives for combatting antimicrobial resistance (AMR), strengthening health emergency preparedness and response, and enhancing the overall quality and safety of healthcare delivery.

Hand hygiene (HH) is widely recognized as a cornerstone of infection prevention in healthcare settings; however, the safety of handwashing water in public HCFs is often overlooked [2]. Recent evidence shows that contaminated HHSs can harbor multidrug-resistant organisms, turning a key preventive measure into a potential source of infection [3]. According to the WHO’s “minimum requirements for IPC programmes”, water used for HH should be microbiologically safe (i.e., no detectable *Escherichia coli* [*E. coli*] in 100 mL and/or a free chlorine residual (FCR) of at least 0.5 mg/L) to prevent direct and indirect exposure to waterborne pathogens from enteric and environmental sources [4]. Ideally, safe water should be available across all clinical areas, but at a minimum, it must be ensured in high-risk wards where the incidence of HAIs and AMR is particularly high [4]. The importance of HH for effective IPC and the risks associated with unsafe HHSs underscore the need for rigorous water quality monitoring.

In Latin America, studies have highlighted that poor HH practices are influenced by factors such as inadequate education, lack of resources, and substandard government regulations [5]. We previously conducted direct observation of HH practices among healthcare workers in Quetzaltenango, Guatemala, providing evidence for low HH adherence, with compliance rates below 50% [6]. In 2021, we conducted a mixed-methods study exploring healthcare workers’ perceptions, knowledge, attitudes, and practices related to HH through interviews and a survey in 19 public HCFs [7]. This study identified key barriers to proper HH, including inadequate infrastructure, supply shortages, and knowledge gaps, particularly concerning the primary routes of transmission for HAIs [7]. Additionally, a water quality assessment was performed in these 19 HCFs, showing that 79% (*n* = 15/19) of water samples tested positive for total coliforms, 52% (*n* = 10/19) for *E. coli*, 21% (*n* = 4/19) for presumptive ESBL-producing bacteria, and 5% (*n* = 1/19) for carbapenem-resistant enterobacterales. These findings highlight the need to couple improvements in HH with safe water provision to reduce infection risk.

Water and sanitation systems in Central America typically receive insufficient investments from governmental agencies, including limited support for water treatment and development of water quality monitoring systems, especially in areas of extreme poverty [8]. These deficiencies become apparent when examining water quality outcomes. For instance, studies on the quality of household water across Guatemala have detected *E. coli* in 13–53% of water samples [9,10]. These deficiencies can be overcome through water chlorination, identified as a practical and low-cost method to improve water safety in healthcare and community settings [11]. On-site chlorination systems are highly effective at reducing contamination with coliforms and *E. coli* in water sources [12], as has been demonstrated among communities in Honduras, Guatemala, and Nicaragua [13,14]. Passive disinfection with chlorine tablet-based methods, which are low-maintenance, electricity-free, and easy to construct and maintain, has been successfully accepted by communities in Central America [14]. Because water chlorination itself is not always accepted by communities [15], its implementation requires ethnographic and anthropological approaches to enhance community acceptance, improve management, and promote the long-term sustainability of water treatment interventions [8].

Here, we aimed to assess the effectiveness of on-site chlorine dispensers installed in water tanks of HCFs in reducing coliform, *E. coli*, and presumptive ESBL-producing bacteria in HHSs. We also employed participatory action research principles through focus groups with HCF staff, municipal water managers, and departmental health authorities for the co-design of action plans to support the sustained use of the CDs.

## 2. Materials and Methods

We carried out a water chlorination intervention through the installation of CDs in water tanks of HCFs in the department of Quetzaltenango, located in the Western Highlands of Guatemala. During the year of the implementation (2022), Quetzaltenango had a total of 246 public HCFs that treated a total of 1,354,840 patients at the departmental level. The current project was carried out in four public HCFs (labeled “A” through “D”) located in three municipalities where the approximate number of patients treated in 2022 was as follows: 14,466 at HCF A; 2655 at HCF B; 2685 at HCF C; and 4706 at HCF D. HCFs were eligible for inclusion if (1) water was available, with flow rates deemed sufficient for chlorine release (a hydraulic assessment was conducted by a qualified engineer to verify if the water tank received a minimum inflow sufficient to intermittently wet the chlorine tablets within the chlorine dispensers; chlorine dispensers were installed only in systems meeting this criterion); (2) water could be directed from its source to a water tank and subsequently to HHSs (point of collection); (3) water from the HCF had detectable levels of *E. coli* and total coliforms in our 2021 assessment; (4) the HCF was located in a community where chlorination was accepted by municipal and community leaders; and (5) the HCF’s management was willing to undertake the required infrastructure investments with financial support from municipal or community authorities. Study sites were prioritized based on baseline microbial load (total coliforms and *E. coli*) to allocate resources where disinfection interventions were clinically and operationally imperative based on the 2021 water quality assessment. Conversely, HCFs with water meeting baseline safety standards that required no corrective chlorination were not considered for inclusion.

**Figure 1:**
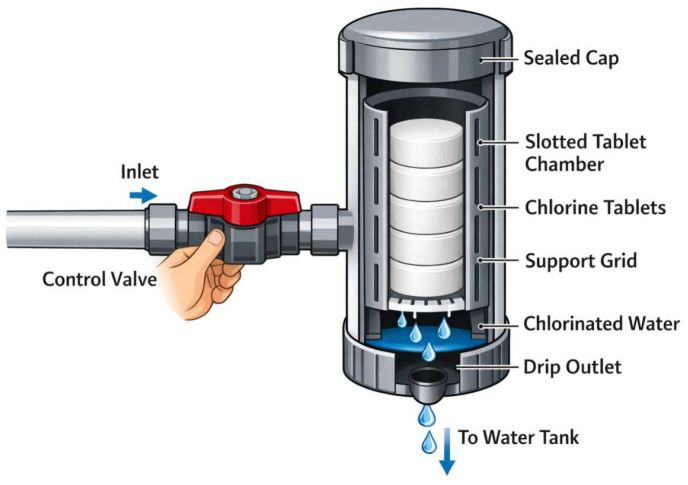
Schematic representation of the chlorine dispenser (created by the authors using AI-assisted illustration tools).

The intervention included the installation of locally manufactured on-site CDs (see Figure 1), followed by weekly water quality testing. The CD employed a slotted tube with concentrated sodium hypochlorite tablets that dissolved as water flows through, releasing about 70% active chlorine. A manual valve regulated the dose, requiring regular monitoring, particularly during the initial setup, to ensure proper performance.

### 2.1. Adaptation of Water Supply Systems

Prior to the installation of CDs, a detailed evaluation was conducted to determine if infrastructure modifications were needed in each HCF. Municipal and community authorities, with the support of “Engineers Without Borders”, adapted water supply systems in August 2022 and January 2023 to facilitate the installation of CDs in April 2023.

### 2.2. Chlorinator Installation and Water Quality Testing

An 8-week baseline assessment (control phase), conducted between February and April 2023, preceded the installation of CDs and included weekly water quality monitoring of HHSs, with one sample collected from each HHS per sampling event. During this period, one sample was collected from each water tank. After installation and calibration of CDs (10 days, April 2023), water quality was monitored weekly for 26 weeks (intervention phase, May–November 2023). One sample was collected from each HHS per sampling event per HCF. From weeks 9 to 26, samples were taken from water tanks to account for potential long-term variation in the chlorination dispensing system.

Weekly water quality monitoring in both the control and intervention phases included testing for the FCR to assess chlorine tablet dissolution and to ensure a consistent chlorine dosage. It also included microbiological water quality testing to detect the presence of coliforms, *E. coli*, and presumptive ESBL-producing bacteria. We selected *E. coli* and total coliforms as indicators of fecal contamination following the WHO’s guidelines for drinking water quality [16]. Although water tanks supplied all HHSs in the facility, the HHS with the highest frequency of use, as identified by HCF staff, was chosen for water quality testing throughout the entire study. High-use stations were prioritized as they represent the main sources of exposure for users.

After installation, the system was calibrated under high-flow conditions and adjusted manually using the control valve. Per Guatemala’s national regulations, drinking water should have a chlorine concentration of 0.5–1.0 mg/L, as 0.5 mg/L is the minimum estimated value for the elimination of pathogenic bacteria [17]. Thus, CD performance was verified by measuring the FCR at the point of use, targeting concentrations between 0.5 and 1.0 mg/L in line with the national regulations. To measure the FCR concentrations, water sources were flushed for 15 s before a sample was collected, which was then analyzed using a Hach^®^ Color Disk Test Kit (Loveland, CO, USA).

For the microbiological water quality analysis, we originally aimed to only culture water samples from HHSs if the FCR concentration was ≤0.2 mg/L. This concentration was established as the cutoff for microbiological testing as it inhibits the growth of bacteria such as *E. coli* by making them non-culturable, without necessarily killing them [18]. During the first 8 weeks of the 26-week intervention phase, all samples were above this threshold, and thus no cultures were taken during this period. To eliminate potential selection bias and ensure comprehensive microbiological surveillance, the protocol was adjusted from week 9 of the intervention phase onwards to culture all samples from water tanks and from HHSs regardless of the FCR concentration to ensure that the microbiological outcomes were directly assessed rather than inferred from the FCR. In addition to the FCR, the pH of the water was monitored using semi-quantitative test strips.

To assess microbial contamination, water (100 mL) was collected into sterile bottles containing sodium thiosulfate to neutralize any existing chlorine. Sampling was conducted at least 30 min after tablet replacement, following the WHO’s guidelines to allow for adequate chlorine contact time [1]; however, contact time was not directly quantified (i.e., no CT calculation or hydraulic assessment was performed). Samples were transported on ice and assayed within 12 h using IDEXX Colilert^®^-18 Quanti-Tray^®^/2000 reagents (Westbrook, Maine) utilizing Defined Substrate Technology following the manufacturer’s instructions for the detection of coliforms and *E. coli*. This method provides quantitative results based on the most probable number (MPN) approved by U.S. EPA and recognized under ISO 9308-2_2012 standards. For each batch of samples, a negative control (sterile water) and a positive control (*E. coli* ATCC^®^ 25922) were processed. If the sample results were positive for either *E. coli* or coliforms (other than *E. coli*), the samples were streaked onto CHROMagar™ (Paris, France) ESBL for the detection of presumptive ESBL-producing bacteria, identified by phenotypic screening without confirmatory molecular characterization. Any detection of *E. coli* in 100 mL was classified as non-compliant with the WHO’s standards [12].

Installation and maintenance of the CDs and weekly water quality testing were conducted by the study team. Training in operation and maintenance of CDs was provided to HCF staff to promote the continuity of the intervention following the end of the study.

### 2.3. Outcomes

The primary outcomes of this study were the proportion of water samples positive for (i) total coliforms, (ii) *E. coli*, and (iii) presumptive ESBL-producing bacteria.

### 2.4. Statistical Analysis

For each outcome, proportions and respective confidence intervals were calculated as the number of positive samples divided by the total number of samples collected in each phase. Differences in proportions between the control and intervention phases were evaluated using Fisher’s exact test due to small cell counts.

### 2.5. Design of Participatory Action Plans for the Sustainability of the Water Chlorination Intervention

As part of their routine responsibilities, HCF staff monitor the quality of the microbiological source water by measuring the FCR. However, the actual chlorination process is carried out by municipal and/or community authorities. To strengthen these existing efforts, we employed participatory action research principles to identify critical activities and key actors necessary to ensure the continuous delivery of safe water and sanitation services and to incorporate these into an action plan. Four focus groups were conducted from May to August of 2023 with key stakeholders involved in water supply and quality monitoring at HCFs. Across all four HCFs where the water treatment intervention took place, three working groups were organized, each representing a different municipality. HCF representatives, departmental authorities of the Ministry of Public Health and Social Assistance, and municipal water supply managers were invited to participate. All sessions were conducted in person and groups were organized by the municipal health district.

As part of the focus groups, key stakeholders in water supply management were identified (Table 1). Participants identified past water and sanitation interventions conducted at the four HCFs and defined keywords related to sustainability. They also discussed scenarios that would facilitate the long-term continuity of the water chlorination intervention and identified challenges and opportunities for achieving this goal. Actions needed to address the identified challenges were also determined and prioritized. Findings were summarized and integrated into action plans.

To facilitate interpretation and cross-context applicability of the participatory findings, themes that emerging inductively from the focus groups were retrospectively mapped onto the domains of the Consolidated Framework for Implementation Research [19]. This post hoc analytical step was not used to guide data collection but rather to organize and communicate emergent findings within a recognized implementation of science vocabulary.

## 3. Results

### 3.1. Installation of Chlorine Dispensers

**Figure 2:**
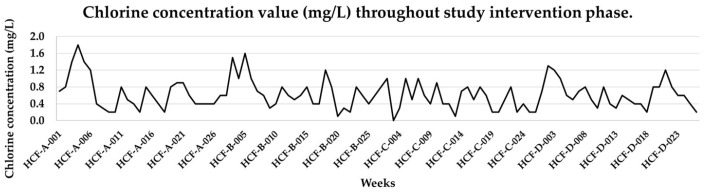
Summary of weekly chlorine concentrations during the 26-week intervention phase in hand hygiene station water samples. Each healthcare facility (HCF) was sampled once per week for 26 weeks. Facilities are labeled as A–D. Sample identifiers on the x-axis follow the format HCF-[facility]-[time], where HCF denotes the healthcare facility, the letter (A–D) indicates the specific facility, and the numeric code (001–026) corresponds to the sampling week (001 = week 1, 002 = week 2, …, 026 = week 26). Each facility therefore contributed 26 samples (total *n* = 104).

Four HCFs located within three different municipalities of the Quetzaltenango Department met the inclusion criteria and were invited to participate. The required infrastructure modifications included redirecting the piped water to the tank and then to the HHS. In some HCFs, installation of stopcocks to control the flow rate entering the water tanks was also needed. After installation of the CDs, chlorine levels were monitored daily for ten days to calibrate the concentration recommended by national regulations (0.5–1.0 mg/L). However, variations in water flow and mechanical failures reported during the calibration period resulted in the observed FCR ranging from 0.1 to 2.8 mg/L (throughout this period, staff were instructed not to use the HCF water systems, and an alternative water supply was provided). During the intervention phase, the FCR ranged from 0 to 1.8 mg/L. Figure 2 summarizes the chlorine concentrations reported over the 26-week intervention phase. Values were subsampled (every fifth measurements) to facilitate visualization. Water pH remained stable at 6.0 throughout all measurements across the control and intervention phases.

### 3.2. Water Quality Testing

During the control phase, a total of 40 water samples were taken from HHSs and 5 samples from water tanks. All 45 samples were cultured as their chlorine concentrations were below 0.2 mg/L. During the intervention phase, a total of 104 water samples were collected from HHSs and 90 from water tanks. During the first 8 weeks of the intervention phase, no samples were cultured because the FCR exceeded 0.2 mg/L. In the subsequent 18 weeks, all samples were cultured. The analysis was restricted to the final 18 weeks of monitoring, when samples were cultured irrespective of the FCR.

During the control phase, 75% (95% CI: 60–86%; *n* = 30/40) of HHS samples tested positive for total coliforms, of which 50% (95% CI: 33–67%; *n* = 15/30) were presumed to be ESBL-producing. During the intervention phase, total coliforms decreased to 1% (*n* = 1/72; *p* < 0.001), and no presumptive ESBL-producing total coliforms were detected.

Similarly, *E. coli* decreased from 20% (95% CI: 11–35%; *n* = 8/40) during the control phase to 0% (*n* = 0/72; *p* < 0.001) during the intervention phase. Accordingly, presumptive ESBL-producing *E. coli* decreased from 50% (95% CI: 11–35%; *n* = 4/8) to 0% with no positive isolates detected during the intervention phase.

No statistically significant differences were observed for any outcomes regarding the tanks’ water during the control and intervention phase (all *p* > 0.05; see Supplementary Materials).

### 3.3. Results of Participatory Action Plans for the Sustainability of the Water Chlorination Intervention

Four focus groups were conducted with key stakeholders across three municipalities with a total of 22 participants. Table 2 summarizes the themes that emerged from the focus groups, mapped to the domains of the CFIR.

The key governmental, municipal, and community informants identified differences in the importance and responsibility placed on water supply management. Differences were noted by municipality. For instance, in one municipality, community leaders oversaw water supply and maintenance, whereas in the other two, these tasks fell under the jurisdiction of municipal authorities. In the latter two communities, the nature of support from municipal authorities differed greatly. For instance, in one municipality, water system support had been consistent over the years, while in the other, it had not.

Despite the contextual differences in each HCF, common challenges were identified concerning ensuring the sustainability of the chlorination intervention. Participants anticipated future difficulties in securing the financial, material, and human resources necessary for the proper maintenance of chlorine dispensers. Additionally, they highlighted the need to work with staff to increase capacity to effectively monitor water quality and CD function. Participants emphasized administrative and bureaucratic processes at the governmental and municipal levels that delayed the procurement of materials including chlorine tablets and the equipment for CD maintenance, requiring unanticipated purchases that were unlikely to be accommodated by the government.

One common opportunity suggested by participants was the development of a comprehensive operational manual to ensure the sustainability of water chlorination intervention. Participants suggested that this manual should outline key actors, responsibilities, resources, timelines, and specific guidelines for the maintenance of chlorine dispensers at each HCF. Strengthening relationships between governmental, municipal, and community institutions was also identified as a key opportunity to enhance access to essential resources. Formation of alliances with external stakeholders, such as non-governmental organizations and universities, was highlighted as a valuable strategy to secure resources that are often difficult to obtain, including supplies and technical support.

The primary strategy identified for ensuring sustainability was resource acquisition and maintenance support through the organization of communities. Participants suggested that sharing successful experiences among HCFs could help gain the support of community leaders and initiate dialog in areas where significant communication barriers persist.

These discussions, covering stakeholders, roles, responsibilities, challenges, and opportunities for chlorine dispenser sustainability, informed each municipality’s participant-designed action plan. The final action plans integrated objectives, specific actions, responsible parties, timeframes, resources, success indicators, and anticipated problems with solutions. These plans were subsequently delivered to municipal health coordinators and the Quetzaltenango Health Directorate authorities, key actors in monitoring their adaptation and implementation.

## 4. Discussion

### 4.1. Effectiveness of the Installation of On-Site Chlorine Dispensers in Improving Handwashing Water Quality in Healthcare Facilities

This study demonstrated the effectiveness of an on-site chlorine dispenser to reduce the presence of total coliforms, *E. coli*, and presumptive ESBL-producing bacteria in hand hygiene water used by healthcare providers. The absence of total coliforms, *E. coli*, and presumptive ESBL-producing bacteria in samples taken during the intervention phase supports the effectiveness of the chlorine dispenser. The absence of changes in outcomes at the water tank level is consistent with the intervention design and supports the interpretation that observed improvements were attributable to the intervention rather than changes in source water quality. Water pH, measured using semi-quantitative strips, remained within acceptable ranges throughout, suggesting minimal impact on bacterial recovery or outcomes. At the observed pH level, free chlorine residual exists predominantly as hypochlorous acid (HOCl), the most potent biocidal form, which optimizes the disinfection kinetics achieved during 30 min of contact time.

Systems that do not require a constant flow rate (as chlorine release is governed by intermittent wetting of the tablet surface rather than continuous dissolution) allow for CDs to function under variable hydraulic conditions, which are common in low-resource settings. This approach increases the applicability of the system in real-world conditions where flow fluctuations are unavoidable. Despite this, variability in flow may influence chlorine release rates, which underscores the importance of periodic monitoring and manual calibration to maintain the FCR within the recommended limits.

Similar devices have primarily been installed in community settings rather than in on-site water tanks at the facility level; therefore, comparability with other HCF-based interventions is limited [13]. Passive chlorinators have been used in rural Tanzania to provide safe drinking water in HCFs within cholera hotspots [20]. Our findings demonstrate that in settings where water management systems or supply chains do not reliably provide chlorinated water to HCFs, CDs can provide safe hand hygiene water. Furthermore, as these CDs are contained within HCFs and water quality monitoring occurs on-site, sustainability and maintenance may be more feasible compared to CDs installed in larger municipal systems. Additionally, the smaller and more localized scale of operation likely allows for easier oversight by HCF staff. Scaling up this intervention introduces challenges for sustainability and maintenance, highlighting the need for active involvement of actors from multiple sectors.

### 4.2. Sustainability of the Water Chlorination Intervention

The participatory approach of this intervention provided a nuanced understanding of the technical and structural contexts specific to each HCF to ensure sustainability. The key actors identified in water supply management and quality monitoring varied between municipalities, as expected, given the structural differences identified in water supply and treatment practices at each HCF. We attribute this variance to municipalities operating in silos, unaccustomed to exchanging ideas regarding water safety across sectors and communities. Within this fragmented context, municipal governments are the primary entities responsible for providing drinking water and sanitation services at the municipal and community levels. To fulfill this role, they receive technical, financial, and administrative support from the Municipal Development Institute, an autonomous state institution. Further, water and sanitation oversight is provided by the Ministry of Health, and organized community committees actively participate in ensuring safe drinking water. This governance framework is grounded in decentralization policies and participatory planning processes aimed at improving the coverage and quality of these services. However, challenges remain regarding the clear assignment of responsibilities and effective coordination among the various actors involved [17].

Successful implementation of chlorination interventions requires discussion about standardized action plans, specifically between municipal and community authorities, and facilitated conversations to identify common challenges, opportunities, and strategies for the long-term success of water chlorination. Through these conversations, financial and technical barriers toward implementation were identified and are consistent with challenges reported during chlorination interventions in other low- and middle-income countries [13,21]. Here, cross-sector communication was facilitated because of the participatory components of the intervention, and materials will be provided to HCFs to overcome these challenges. Comprehensive management plans addressing the entire water supply while contextualizing water treatment interventions can improve sustainability [1,22]. To our knowledge, however, this is the first publication of cross-sectoral implementation to ensure an HCF’s ability to provide safe hand hygiene water to healthcare providers in Guatemala.

Our ethnographic experience revealed that although the Ministry of Health is responsible for water quality monitoring, the department of Quetzaltenango faces significant limitations, including inadequate infrastructure, limited personnel, and scarce financial resources. Conducting detailed water analyses requires sending samples to Guatemala City, presenting additional logistical and operational challenges to maintaining a consistent and safe water supply in healthcare settings.

### 4.3. Limitations

Water quality testing revealed that the chlorine dispensers were highly effective at eliminating total coliforms, *E. coli*, and presumptive ESBL-producing bacteria. However, these findings should be interpreted considering the following limitations. Water quality monitoring was limited to the most frequently used hand hygiene stations at each HCF, which may not have captured variability across other points in the system. Nevertheless, the shared chlorinated water source suggests that the findings may be representative of overall system disinfection performance. The change in culturing criteria during the intervention period—initially limited to samples with chlorine concentrations below 0.2 mg/L and later expanded to all samples—may have introduced selection bias and should be considered when interpreting the findings. Additionally, neither water turbidity, temperature, nor chlorine contact time were formally measured. Thus, variability in chlorine efficacy cannot be fully accounted for, limiting the interpretation of disinfection performance. Furthermore, we only included HCFs with high baseline contamination as a targeted approach to generate evidence in settings with water quality failures and significant public health needs. However, given the limited sample size, regression to the mean cannot be excluded as a partial explanation for the observed reductions. The fact that the identification of presumptive ESBL-producing bacteria was based on phenotypic screening without confirmatory molecular characterization limits interpretation of the intervention’s effectiveness against antimicrobial-resistant organisms. Nevertheless, chromogenic agar remains a widely accepted tool for environmental surveillance.

Inconsistent participation by municipal authorities in focus groups impeded the intersectoral dialog necessary for co-designing action plans fully grounded in each stakeholder’s specific roles. Additionally, limited participant availability constrained the depth and robustness of the plans, as developing sustainable proposals requires ongoing, long-term engagement. Although four workshops were held, the timeframe was insufficient to refine the plans, conduct a thorough validation process, or involve other key stakeholders in water management for HCF. These challenges underscore the importance of securing stronger institutional commitments and allowing more time for future participatory processes. And finally, it is important to highlight that the focus group discussions centered on the sustainability of the CDs installed in the four participating HCFs, which directed the scope of the dialogue. Consequently, issues related to laboratory processing of water samples were not explored.

## 5. Conclusions

This study demonstrates the effectiveness of on-site chlorine dispensers in improving hand hygiene water quality in HCFs by eliminating total coliforms, *E. coli*, and presumptive ESBL-producing bacteria through a consistent chlorine dosage. The development of integrated action plans with key stakeholders across community, municipal, and government sectors emphasized the need to facilitate cross-sector relationships to ensure the long-term sustainability of chlorination interventions at HCFs. These findings offer a structured basis for replication and adaptation of this model in comparable low-resource settings.

## Figures and Tables

**Table 1:** Focus group sessions for the participatory action planning process.

Objectives	Methods	Output
Present project and baseline water quality results; identify past water interventions; map key actors and responsibilities.	PowerPoint presentation; free listing; stakeholder mapping.	Stakeholder maps and inventory of prior water interventions per municipality.
Define sustainability; identify facilitating scenarios for long-term water chlorination continuity; analyze barriers and opportunities.	Flow diagram; balloons and stones exercise.	Shared sustainability definitions; barrier–opportunity matrices per municipality.
Recap of focus group 1–2 findings; identify and prioritize sustainability strategies.	Free listing; card sorting (by importance, urgency, and feasibility).	Prioritized action lists per municipality; preliminary action plan inputs.
Update on water monitoring results; co-design sustainability action plans; establish sectoral commitments.	SMART action planning; timeline planning; exit survey.	Finalized action plans and sustainability timelines delivered to municipal and departmental health authorities.

**Table 2:** Participatory findings mapped to the Consolidated Framework for Implementation Research (CFIR).

CFIR Domain	Barrier Identified	Opportunity Identified	Action Plan Component
Intervention characteristics	Mechanical failures and variable water flow compromised CD performance; maintenance requires unanticipated purchases outside routine budgets.	Small, localized scale of HCF-based dispensers allows for easier oversight than municipal systems; low-cost design is replicable.	Develop an operational manual specifying CD calibration protocols, maintenance schedule, and contingency steps for mechanical failures.
Outer setting (policies and governance)	Administrative and bureaucratic delays at governmental and municipal levels impede procurement of chlorine tablets and maintenance supplies.	Existing decentralization policies and community water committees provide governance infrastructure to anchor sustainability.	Map procurement pathways; engage municipal health coordinators and the Quetzaltenango Health District as official oversight actors.
Inner setting (organizational capacity)	Limited financial, material, and human resources within HCFs; inconsistent staff capacity to monitor water quality and dispenser function.	HCF staff have direct stake in safe water and were trained in dispenser operation during the study period.	Include staff roles, responsibilities, and resource requirements in operational manual; build in capacity-strengthening activities.
Inner setting (inter-organizational relations)	Municipalities operate in silos; fragmented coordination among HCF staff, municipal water managers, and Ministry of Health personnel.	Participatory process facilitated cross-sector dialog; shared experiences across HCFs can strengthen collective identity.	Strengthen inter-institutional relationships; create a shared communication platform across the three municipalities.
Characteristics of individuals	Inconsistent participation of municipal authorities in focus groups limited co-design depth and intersectoral agreement.	Key informants varied productively across municipalities, reflecting diverse governance models (community- vs. municipality-led).	Identify and formally assign a sustainability champion within each HCF and municipality; secure institutional commitment from leadership.
Implementation process (external partnerships)	Insufficient long-term engagement time: four workshops were insufficient to fully refine and validate action plans.	Formation of alliances with NGOs, universities, and Engineers Without Borders to secure technical support and supplies.	Formalize alliances with external stakeholders; incorporate plan into multi-year sustainability agreements with defined timeframes and success indicators.

## Data Availability

The dataset(s) supporting the conclusions of this article may be provided upon request to the corresponding author.

## Supplementary Materials

The following supporting information can be downloaded at https://www.mdpi.com/article/10.3390/tropicalmed11050111/s1. Table S1: Codes assigned to handwashing stations and water tanks at each healthcare facility; Table S2: Assigned risk levels for water tanks and handwashing stations in healthcare facilities included in the study; Table S3: Most probable number (MPN) of total coliforms and *E. coli*, and detection of presumptive ESBL-producing bacteria in water samples from hand hygiene stations collected during the control phase: Table S4: Most probable number (MPN) of total coliforms and *E. coli*, and detection of presumptive ESBL-producing bacteria in water samples from tanks collected during the control phase; Table S5: Minimum and maximum values of free residual chlorine detected at hand hygiene stations in healthcare facilities during the intervention phase; Table S6: Most probable number (MPN) of total coliforms, *E. coli*, and presumptive ESBL-producing bacteria in tank water samples (weeks 9–26, intervention phase); Table S7: Free residual chlorine levels detected at handwashing stations in healthcare facilities during the chlorine dispenser installation phase; Table S8: Free chlorine residual values detected at hand hygiene stations in healthcare facilities during the intervention phase. Table S9: Prevalence and 95% confidence intervals of total coliforms, presumptive ESBL-producing total coliforms, *E. coli*, and presumptive ESBL-producing *E. coli* in tank water during control and intervention phases.

## Abbreviations

The following abbreviations are used in this manuscript:

AMR	Antimicrobial resistance
CDC	Centers for Disease Control and Prevention
CRE	Carbapenem-resistant Enterobacterales
*E. coli*	*Escherichia coli*
ESBL	Extended-spectrum beta-lactamase
HAIs	Healthcare-associated infections
HCFs	Healthcare facilities
HH	Hand hygiene
IPC	Infection prevention and control
WASH	Water, sanitation, and hand hygiene
WHO	World Health Organization
