# Supplementary material for: Effectiveness and Sustainability of Water Chlorination in Public Healthcare Services in Guatemala
Source: Trop Med Infect Dis. 2026 Apr 23;11(5):111. doi: 10.3390/tropicalmed11050111 (PMC13211354; doi:10.3390/tropicalmed11050111)
Supplement: Supplementary file 1 [file tropicalmed-11-00111-s001.zip › tropicalmed-4208079-supplementary.pdf]

Tropical Medicine and Infectious Disease

MDPI

Supplementary Materials

This document presents the results of water sampling conducted at the main handwashing stations (HWS) of participating healthcare facilities (HCFs) during the control, installation, and intervention phases of the study.

Coding of HWS and water tanks by HCF was done, with each assigned a code, as shown in Table S1 below.

Table S1. Codes assigned to hand hygiene stations and water tanks at each healthcare facility.

HCF	HWS	Tank*
**HCF A (HCF2)**	**HCF2-RIL1**	**HCF2-RIT1**
**HCF B (HCF3)**	**HCF3-RIL1**	**HCF3-RIT1**
**HCF C (HCF4)**	**HCF4-RIL1**	**HCF4-RIT1**
**HCF D (HCF5)**	**HCF5-RIL1**	**HCF5-RIT1**
***When the HCF had more than one tank, such as HCF4, it was coded: HCF4-RIT1, HCF4-RIT2.**		

Table S2 summarizes the risk levels assigned to each HWS and water tank in the five HCF included in the study. Based on WHO criteria from the Water and Sanitation for Healthcare Facility Improvement Tool (15), scores were used to classify water, sanitation, and hygiene safety risk as follows: low (0–2), medium (3–5), high (6–8), and very high (9–10).

Table S2. Assigned risk levels for water tanks and handwashing stations in healthcare facilities included in the study.

HCF	Risk level	
	Tanks	HWS
HCF 1	Mid (1st evaluation)	Low
HCF 2	Mid (3rd evaluation)	
HCF 3	Low (1st evaluation)	
	Mid (2nd and 3rd evaluation)	
HCF 4	Mid (1st evaluation)	
	Low (2nd and 3rd evaluation)	
HCF 5	High risk (2nd evaluation)	
	Mid (3rd evaluation)	

Total coliforms were detected in 75% (30/40) of the HWS samples, and among these, 50% (15/30) tested positive for presumptive ESBL-producing bacteria. Similarly, *E. coli* was detected in 20% (8/40) of the total samples, and among these, 50% (4/8) also showed the presence of ESBL-producing bacteria (see Table S3).

Table S1. Most probable number (MPN) of total coliforms and *E. coli*, and detection of presumptive ESBL-producing bacteria in water samples from hand hygiene stations collected during the control phase.

HCF	HWS	NMP Total coliforms	BLEE Total coliforms	NMP *E. coli*	BLEE (*E. coli*)
HCF2	HCF2_RIL1_C01	16	Negative	<1	NA
HCF2	HCF2_RIL1_C02	<1	NA	<1	NA
HCF2	HCF2_RIL1_C03	6.3	Positive	<1	NA
HCF2	HCF2_RIL1_C04	12.2	Positive	<1	NA
HCF2	HCF2_RIL1_C05	14.6	Positive	4.1	Positive
HCF2	HCF2_RIL1_C06	461.1	Positive	12	Negative
HCF2	HCF2_RIL1_C07	<1	NA	<1	NA
HCF2	HCF2_RIL1_C08	31.3	Negative	<1	NA
HCF3	HCF3_RIL1_C01	26.9	Negative	3	Negative

HCF	HWS	NMP Total coliforms	BLEE Total coliforms	NMP *E. coli*	BLEE (*E. coli)*
HCF3	HCF3_RIL1_C02	21.1	Positive	<1	NA
HCF3	HCF3_RIL1_C03	74.3	Positive	2	Positive
HCF3	HCF3_RIL1_C04	16	Positive	<1	NA
HCF3	HCF3_RIL1_C05	48	Negative	4.1	Positive
HCF3	HCF3_RIL1_C06	<1	NA	<1	NA
HCF3	HCF3_RIL1_C07	69.7	Positive	<1	NA
HCF3	HCF3_RIL1_C08	40.8	Positive	<1	NA
HCF4	HCF4_RIL1_C01	38.1	Positive	<1	NA
HCF4	HCF4_RIL1_C02	15.5	Negative	<1	NA
HCF4	HCF4_RIL1_C03	3.1	Negative	<1	NA
HCF4	HCF4_RIL1_C04	185	Negative	<1	NA
HCF4	HCF4_RIL1_C05	21.3	Negative	1	Negative
HCF4	HCF4_RIL1_C06	<1	NA	<1	NA
HCF4	HCF4_RIL1_C07	<1	NA	<1	NA
HCF4	HCF4_RIL1_C08	67	Negative	<1	NA
HCF5	HCF5_RIL1_C01	<1	NA	<1	NA
HCF5	HCF5_RIL1_C02	2	Negative	<1	NA
HCF5	HCF5_RIL1_C03	<1	NA	<1	NA
HCF5	HCF5_RIL1_C04	<1	NA	<1	NA
HCF5	HCF5_RIL1_C05	3	Negative	<1	NA
HCF5	HCF5_RIL1_C06	<1	NA	<1	NA
HCF5	HCF5_RIL1_C07	<1	NA	<1	NA
HCF5	HCF5_RIL1_C08	10.7	Negative	<1	NA

Table S4 presents the Most Probable Number (MPN) results for total coliforms and *Escherichia coli*, as well as the presence of presumptive ESBL-producing bacteria from water samples collected during the control phase from the water tanks supplying each health facility. All water tank samples (5/5) were positive for total coliforms, with 40% (2/5) showing presumptive ESBL-producing coliforms. Only one sample tested positive for *E. coli*, and it was negative for presumptive ESBL.

Table S4. Most probable number (MPN) of total coliforms and *E. Coli*, and detection of ESBL-producing bacteria in water samples from tanks collected during the control phase.

HCF	Tank	MPN Total Coliforms	ESBL (Total Coliforms)	MPN *E. coli*	ESBL (*E. coli)*
HCF2	HCF2_RIT1_C01	7.5	Positive	<1	NA
HCF3	HCF3_RIT1_C01	35.9	Positive	4.1	Negative
HCF4	HCF4_RIT1_C01	80.1	Negative	<1	NA
HCF4	HCF4_RIT2_C01	2.0	Negative	<1	NA
HCF5	HCF5_RIT1_C01	4.1	Negative	<1	NA

Table S5 presents the results of free chlorine residual measurements in water samples collected during the intervention phase from HHS in the participating HCF, highlighting the minimum and maximum values.

Table S2. Minimum and maximum values of free chlorine residual detected at hand hygiene stations in healthcare facilities during the intervention phase.

HWS	Minimum chlorine value (mg/L)	Maximum chlorine value (mg/L)
HCF2_RIL1	0.2	1.8
HCF3_RIL1	0	1.6
HCF4_RIL1	0.1	1.0
HCF5_RIL1	0.2	1.3

Table S6 presents the Most Probable Number (MPN) of total coliforms and *E. coli*, and the presence of presumptive ESBL-producing bacteria from total coliforms and *E. coli*, respectively, in water samples collected at the tank inlet before chlorination from week nine to week twenty-six during the intervention phase in the water tanks supplying the participating health care facilities.

Table S3. Most probable number (MPN) of total coliforms and *E. Coli*, and ESBL-producing bacteria in tank water samples (Weeks 9–26, intervention phase).

Tank code	MPN Total coliforms	ESBL Total coliforms	MPN *E. coli*	ESBL *E. coli*
HCF2_RIT1_F09	77.1	Negative	<1	NA
HCF2_RIT1_F10	115.3	Positive	<1	NA
HCF2_RIT1_F11	98.7	Negative	2	Negative
HCF2_RIT1_F12	101.9	Positive	<1	NA
HCF2_RIT1_F13	<1	NA	<1	NA
HCF2_RIT1_F14	218.7	Positive	16.5	Positive
HCF2_RIT1_F15	209.8	Negative	<1	NA
HCF2_RIT1_F16	11	Positive	<1	NA
HCF2_RIT1_F17	40.2	Positive	<1	NA
HCF2_RIT1_F18	77.6	Negative	1	Negative
HCF2_RIT1_F19	<1	NA	<1	NA
HCF2_RIT1_F20	275.5	Positive	16.9	Positive
HCF2_RIT1_F21	54.5	Positive	3	Negative
HCF2_RIT1_F22	365.4	Negative	1	Negative
HCF2_RIT1_F23	1986.3	Positive	4.1	Negative
HCF2_RIT1_F24	387.3	Negative	5.2	Negative
HCF2_RIT1_F25	328.2	Negative	1	Negative
HCF2_RIT1_F26	108.1	Negative	4.1	Negative
HCF3_RIT1_F09	49.6	Negative	2	Negative
HCF3_RIT1_F10	67	Negative	1	Negative
HCF3_RIT1_F11	235.9	Negative	4.1	Negative
HCF3_RIT1_F12	95.6	Negative	<1	NA
HCF3_RIT1_F13	166.4	Negative	1	Negative
HCF3_RIT1_F14	142.1	Negative	6.1	Negative
HCF3_RIT1_F15	579.4	Negative	3.1	Negative
HCF3_RIT1_F16	115.3	Negative	<1	NA
HCF3_RIT1_F17	648.8	Negative	45.9	Negative
HCF3_RIT1_F18	488.4	Negative	22.8	Negative
HCF3_RIT1_F19	201.4	Positive	21.8	Negative
HCF3_RIT1_F20	290.9	Negative	7.5	Negative
HCF3_RIT1_F21	107.1	Negative	2	Negative
HCF3_RIT1_F22	172.2	Negative	18.3	Negative
HCF3_RIT1_F23	104.3	Negative	8.5	Negative
HCF3_RIT1_F24	187.2	Positive	1	Positive
HCF3_RIT1_F25	84.2	Negative	<1	NA
HCF3_RIT1_F26	146.7	Negative	<1	NA
HCF4_RIT1_F09	<1	NA	<1	NA
HCF4_RIT1_F10	5.2	Negative	1	Negative
HCF4_RIT1_F11	488.4	Negative	6.2	Negative
HCF4_RIT1_F12	116.5	Positive	7.1	Negative
HCF4_RIT1_F13	488.4	Positive	3.1	Positive
HCF4_RIT1_F14	5.2	Negative	<1	NA
HCF4_RIT1_F15	12.2	Negative	<1	NA
HCF4_RIT1_F16	<1	NA	<1	NA

Tank code	MPN Total coliforms	ESBL Total coliforms	MPN *E. coli*	ESBL *E. coli*
HCF4_RIT1_F17	185	Positive	2	Positive
HCF4_RIT1_F18	<1	NA	<1	NA
HCF4_RIT1_F19	248.9	Positive	2	Negative
HCF4_RIT1_F20	88.2	Positive	1	Positive
HCF4_RIT1_F21	123.6	Negative	4.1	Negative
HCF4_RIT1_F22	<1	NA	<1	NA
HCF4_RIT1_F23	7.1	Positive	2	Negative
HCF4_RIT1_F24	64	Negative	<1	NA
HCF4_RIT1_F25	1046.2	Negative	35.9	Positive
HCF4_RIT1_F26	91	Negative	1	Positive
HCF4_RIT2_F09	3.1	Positive	1	Negative
HCF4_RIT2_F10	88.2	Negative	5.2	Negative
HCF4_RIT2_F11	648.8	Positive	29.2	Negative
HCF4_RIT2_F12	66.3	Negative	4.1	Negative
HCF4_RIT2_F13	272.3	Positive	<1	NA
HCF4_RIT2_F14	<1	NA	<1	NA
HCF4_RIT2_F15	24.1	Positive	1	Positive
HCF4_RIT2_F16	<1	NA	<1	NA
HCF4_RIT2_F17	191.8	Positive	1	Positive
HCF4_RIT2_F18	<1	NA	<1	NA
HCF4_RIT2_F19	210.5	Positive	6.3	Negative
HCF4_RIT2_F20	547.5	Negative	<1	NA
HCF4_RIT2_F21	>2419.6	Negative	34.1	Negative
HCF4_RIT2_F22	2	Positive	<1	NA
HCF4_RIT2_F23	1	Positive	<1	NA
HCF4_RIT2_F24	83.6	Positive	<1	NA
HCF4_RIT2_F25	344.8	Negative	6.3	Negative
HCF4_RIT2_F26	114.5	Positive	3.1	Positive
HCF5_RIT1_F10	6.3	Negative	<1	NA
HCF5_RIT1_F11	11	Negative	<1	NA
HCF5_RIT1_F12	18.5	Negative	1	Negative
HCF5_RIT1_F13	93.3	Negative	<1	NA
HCF5_RIT1_F14	37.4	Negative	<1	NA
HCF5_RIT1_F15	12.1	Negative	<1	NA
HCF5_RIT1_F16	35.9	Positive	<1	NA
HCF5_RIT1_F17	12.1	Negative	<1	NA
HCF5_RIT1_F18	9.7	Negative	<1	NA
HCF5_RIT1_F19	21.6	Negative	<1	NA
HCF5_RIT1_F20	11	Negative	<1	NA
HCF5_RIT1_F21	<1	NA	<1	NA
HCF5_RIT1_F22	<1	NA	<1	NA
HCF5_RIT1_F23	10.9	Negative	<1	NA
HCF5_RIT1_F24	33.1	Negative	<1	NA
HCF5_RIT1_F25	3.1	Negative	<1	NA
HCF5_RIT1_F26	3.1	Negative	<1	NA
HCF5_RIT1_F27	<1	NA	<1	NA

None of the water samples collected from the hand hygiene stations during the chlorine dispenser installation phase were microbiologically analyzed. Chlorine levels ranged from 0.1 to 2.8 mg/L. Table S7 presents the results of all chlorine measurements.

Table S7. Free residual chlorine levels detected at handwashing stations in healthcare facilities during the chlorine dispenser installation phase.

HWS code	Chlorine concentration (mg/L)
HCF2_RIL1_I01	1.8
HCF2_RIL1_I02	2.0
HCF2_RIL1_I03	2.8
HCF2_RIL1_I04	1.0
HCF2_RIL1_I05	0.8
HCF2_RIL1_I06	0.6
HCF2_RIL1_I07	0.7
HCF2_RIL1_I08	0.9
HCF2_RIL1_I09	0.6
HCF2_RIL1_I10	0.6
HCF3_RIL1_I01	0.1
HCF3_RIL1_I02	2.0
HCF3_RIL1_I03	2.6
HCF3_RIL1_I04	1.7
HCF3_RIL1_I05	0.2
HCF3_RIL1_I06	0.5
HCF3_RIL1_I07	0.2
HCF3_RIL1_I08	0.9
HCF3_RIL1_I09	0.5
HCF3_RIL1_I10	0.6
HCF4_RIL1_I01	0.5
HCF4_RIL1_I02	0.4
HCF4_RIL1_I03	0.4
HCF4_RIL1_I04	0.2
HCF4_RIL1_I05	0.2
HCF4_RIL1_I06	2.0
HCF4_RIL1_I07	0.6
HCF4_RIL1_I08	0.6
HCF4_RIL1_I09	0.8
HCF4_RIL1_I10	1.0
HCF5_RIL1_I01	0.5
HCF5_RIL1_I02	0.4
HCF5_RIL1_I03	0.4
HCF5_RIL1_I04	0.2
HCF5_RIL1_I05	0.2
HCF5_RIL1_I06	0.8
HCF5_RIL1_I07	2.0
HCF5_RIL1_I08	0.6
HCF5_RIL1_I09	0.8
HCF5_RIL1_I10	0.6

Table S8. Free chlorine residual values detected at hand hygiene stations in health care facilities during the intervention phase.

HWS	Chlorine concentration value (mg/L)
HCF2_RIL1_F01	0.7
HCF2_RIL1_F02	0.8
HCF2_RIL1_F03	1.4
HCF2_RIL1_F04	1.8
HCF2_RIL1_F05	1.4
HCF2_RIL1_F06	1.2

HWS	Chlorine concentration value (mg/L)
HCF2_RIL1_F07	0.4
HCF2_RIL1_F08	0.3
HCF2_RIL1_F09	0.2
HCF2_RIL1_F10	0.2
HCF2_RIL1_F11	0.8
HCF2_RIL1_F12	0.5
HCF2_RIL1_F13	0.4
HCF2_RIL1_F14	0.2
HCF2_RIL1_F15	0.8
HCF2_RIL1_F16	0.6
HCF2_RIL1_F17	0.4
HCF2_RIL1_F18	0.2
HCF2_RIL1_F19	0.8
HCF2_RIL1_F20	0.9
HCF2_RIL1_F21	0.9
HCF2_RIL1_F22	0.6
HCF2_RIL1_F23	0.4
HCF2_RIL1_F24	0.4
HCF2_RIL1_F25	0.4
HCF2_RIL1_F26	0.4
HCF3_RIL1_F01	0.6
HCF3_RIL1_F02	0.6
HCF3_RIL1_F03	1.5
HCF3_RIL1_F04	1.0
HCF3_RIL1_F05	1.6
HCF3_RIL1_F06	1.0
HCF3_RIL1_F07	0.7
HCF3_RIL1_F08	0.6
HCF3_RIL1_F09	0.3
HCF3_RIL1_F10	0.4
HCF3_RIL1_F11	0.8
HCF3_RIL1_F12	0.6
HCF3_RIL1_F13	0.5
HCF3_RIL1_F14	0.6
HCF3_RIL1_F15	0.8
HCF3_RIL1_F16	0.4
HCF3_RIL1_F17	0.4
HCF3_RIL1_F18	1.2
HCF3_RIL1_F19	0.8
HCF3_RIL1_F20	0.1
HCF3_RIL1_F21	0.3
HCF3_RIL1_F22	0.2
HCF3_RIL1_F23	0.8
HCF3_RIL1_F24	0.6
HCF3_RIL1_F25	0.4
HCF3_RIL1_F26	0.6
HCF4_RIL1_F01	0.8
HCF4_RIL1_F02	1.0
HCF4_RIL1_F03	0.0
HCF4_RIL1_F04	0.3
HCF4_RIL1_F05	1.0
HCF4_RIL1_F06	0.5

HWS	Chlorine concentration value (mg/L)
HCF4_RIL1_F07	1.0
HCF4_RIL1_F08	0.6
HCF4_RIL1_F09	0.4
HCF4_RIL1_F10	0.9
HCF4_RIL1_F11	0.4
HCF4_RIL1_F12	0.4
HCF4_RIL1_F13	0.1
HCF4_RIL1_F14	0.7
HCF4_RIL1_F15	0.8
HCF4_RIL1_F16	0.5
HCF4_RIL1_F17	0.8
HCF4_RIL1_F18	0.6
HCF4_RIL1_F19	0.2
HCF4_RIL1_F20	0.2
HCF4_RIL1_F21	0.5
HCF4_RIL1_F22	0.8
HCF4_RIL1_F23	0.2
HCF4_RIL1_F24	0.4
HCF4_RIL1_F25	0.2
HCF4_RIL1_F26	0.2
HCF5_RIL1_F01	0.7
HCF5_RIL1_F02	1.3
HCF5_RIL1_F03	1.2
HCF5_RIL1_F04	1.0
HCF5_RIL1_F05	0.6
HCF5_RIL1_F06	0.5
HCF5_RIL1_F07	0.7
HCF5_RIL1_F08	0.8
HCF5_RIL1_F09	0.5
HCF5_RIL1_F10	0.3
HCF5_RIL1_F11	0.8
HCF5_RIL1_F12	0.4
HCF5_RIL1_F13	0.3
HCF5_RIL1_F14	0.6
HCF5_RIL1_F15	0.5
HCF5_RIL1_F16	0.4
HCF5_RIL1_F17	0.4
HCF5_RIL1_F18	0.2
HCF5_RIL1_F19	0.8
HCF5_RIL1_F20	0.8
HCF5_RIL1_F21	1.2
HCF5_RIL1_F22	0.8
HCF5_RIL1_F23	0.6
HCF5_RIL1_F24	0.6
HCF5_RIL1_F25	0.4
HCF5_RIL1_F26	0.2

Table S9. Prevalence and 95% confidence intervals of total coliforms, presumptive ESBL-producing total coliforms, *E. coli*, and presumptive ESBL-producing *E. coli* in water tank during control and intervention phases.

Microbiological Parameter	Phase	Positive	Total	Percentage	95% CI
Total coliforms	Control	5	5	100%	48%–100%
Total coliforms	Intervention	79	90	87%	78%–92%
Presumptive ESBL-producing coliforms	Control	2	5	40%	12%–77%
Presumptive ESBL-producing coliforms	Intervention	27	79	34%	25%–45%
E. coli	Control	1	5	20%	4%–62%
E. coli	Intervention	46	90	51%	40%–61%
Presumptive ESBL-producing *E. coli*	Control	0	1	0%	0%–98%
Presumptive ESBL-producing *E. coli*	Intervention	11	46	24%	14%–38%

Disclaimer/Publisher's Note: The statements, opinions and data contained in all publications are solely those of the individual author(s) and contributor(s) and not of MDPI and/or the editor(s). MDPI and/or the editor(s) disclaim responsibility for any injury to people or property resulting from any ideas, methods, instructions or products referred to in the content.
